# Floquet-state cooling

**DOI:** 10.1038/s41598-019-53877-w

**Published:** 2019-11-26

**Authors:** Onno R. Diermann, Martin Holthaus

**Affiliations:** Institut für Physik, Carl von Ossietzky Universität, D-26111 Oldenburg, Germany

**Keywords:** Quantum physics, Statistical physics, thermodynamics and nonlinear dynamics

## Abstract

We demonstrate that a periodically driven quantum system can adopt a quasistationary state which is effectively much colder than a thermal reservoir it is coupled to, in the sense that certain Floquet states of the driven-dissipative system can carry much higher population than the ground state of the corresponding undriven system in thermal equilibrium. This is made possible by a rich Fourier spectrum of the system’s Floquet transition matrix elements, the components of which are addressed individually by a suitably peaked reservoir density of states. The effect is expected to be important for driven solid-state systems interacting with a phonon bath predominantly at well-defined frequencies.

## Introduction

## Objective

In countless situations of basic experimental and theoretical interest, ranging from superconducting charge pumps^1^ over Dirac fermions in graphene coupled to acoustic phonons^2^ and quantum-dot devices^3^ to superconductors under phonon driving^4^, emitters in laser-driven cavities^5^, or few-level systems coupled to transmission lines^6^, one encounters periodically driven quantum systems interacting with their environment^7–22^. Such systems usually adopt a quasistationary state which may depend substantially on that interaction, even to the extent that the dynamics are fully environment-governed^1^. An investigation of a periodically driven dissipative Bose-Hubbard model has demonstrated that the interaction of a driven system with a reservoir can protect the system against heating^23^. In the same spirit, a recent study of steady states of interacting Floquet insulators has confirmed that a heat bath can stabilize certain low-entropy states of periodically driven interacting systems^24^. This is what one may expect on intuitive grounds, since the system can dump the energy absorbed from the drive into the reservoir^25,26^. With the present contribution we direct this line of research into previously unknown territory, and establish a fairly counterintuitive phenomenon: A periodically driven quantum system interacting with a thermal bath can effectively be made even *colder* than the bath it is coupled to, “effectively” meaning here that a Floquet state of a driven-dissipative quantum system can carry much *higher* population than the undriven system’s ground state in thermal equilibrium.

## Model System

To demonstrate the feasibility of such unexpected Floquet-state cooling we employ the model of a harmonic oscillator with a periodically time-dependent spring function *k*(*t*), which often has served as a workhorse in studies of quantum thermodynamics^27–30^. It is given by the Hamiltonian

1 $${H}_{0}(t)=\frac{{p}^{2}}{2M}+\frac{1}{2}k(t){x}^{2},$$

with *M* denoting the mass of the oscillator particle. We take the spring function to be of the form

2 $$k(t)=M{\Omega }_{0}^{2}-M{\Omega }_{1}^{2}\,\cos (\omega t),$$

so that Ω_0_ is the angular frequency of the undriven oscillator, and the frequency Ω_1_ quantifies the driving strength; with this choice the classical equation of motion $M\ddot{\xi }(t)=-\,k(t)\,\xi (t)$ becomes equal to the famous Mathieu equation^31,32^. Provided the parameters Ω_0_/*ω* and Ω_1_/*ω* are chosen such that the Mathieu equation admits stable solutions, the time-dependent Schrödinger equation with the Hamiltonian (1) possesses a complete set of square-integrable Floquet states, that is, of solutions

3 $${\psi }_{n}(x,t)={u}_{n}(x,t)\exp (\,-\,i{\varepsilon }_{n}t/\hslash )$$

with square-integrable Floquet functions *u*_*n*_(*x*,*t*) which acquire the period *T* = 2*π*/*ω* of the drive,

4 $${u}_{n}(x,t)={u}_{n}(x,t+T).$$

These Floquet states (3) have been obtained independently by several authors^33–35^, and been utilized, *e.g*., to describe the quantum dynamics of particles in a Paul trap^36^. The key element entering into their construction are stable classical Floquet solutions

5 $$\xi (t)=\nu (t)\exp ({\rm{i}}\nu t)$$

with a *T*-periodic function *v*(*t*) = *v*(*t* + *T*) and a corresponding characteristic exponent *ν*^31,32^. The latter can be chosen such that it connects continuously to the oscillator frequency Ω_0_ when the drive is switched off, so that Ω_1_ goes to zero^30^. The quasienergies *ε*_*n*_ which accompany the time evolution of the Floquet states (3) then take the form

6 $${\varepsilon }_{n}=\hslash \nu (n+1/2)\,{\rm{mod}}\,\hslash \omega ,$$

where *n* = 0, 1, 2, 3, … is the usual integer oscillator quantum number. If the combination of parameters Ω_0_/*ω* and Ω_1_/*ω* gives rise to unstable solutions of the Mathieu equation, the quasienergy spectrum of the parametrically driven oscillator becomes absolutely continuous^37^ so that the system can absorb an infinite amount of energy from the drive; this case is not being considered here.

Now let us couple this system (1) to an infinite phonon bath modeled by thermally occupied harmonic oscillators with frequencies $\tilde{\omega }$. To this end, we adopt the interaction Hamiltonian^38^

7 $${H}_{{\rm{int}}}=\gamma x\sum _{\tilde{\omega }}\,({b}_{\tilde{\omega }}+{b}_{\tilde{\omega }}^{\dagger }),$$

where the constant *γ* carries the dimension of energy per length, and the operators ${b}_{\tilde{\omega }}$ and ${b}_{\tilde{\omega }}^{\dagger }$ describe, respectively, annihilation and creation processes in the bath. Following the approach pioneered by Breuer *et al*.^39^, the rate Γ_*fi*_ of bath-induced transitions from an initial Floquet state *i* to a final one *f* then is obtained as a sum

8 $${\Gamma }_{fi}=\sum _{\ell }\,{\Gamma }_{fi}^{(\ell )},$$

where the partial rates

9 $${\Gamma }_{fi}^{(\ell )}=\frac{2\pi }{{\hslash }^{2}}{|{V}_{fi}^{(\ell )}|}^{2}N({\omega }_{fi}^{(\ell )})\,J(|{\omega }_{fi}^{(\ell )}|)$$

correspond to the individual Floquet transition frequencies

10 $${\omega }_{fi}^{(\ell )}=({\varepsilon }_{f}-{\varepsilon }_{i})/\hslash +\ell \omega $$

with $\ell =0,\,\pm \,1,\,\pm \,2,\ldots \,$. The quantities ${V}_{fi}^{(\ell )}$ are given by the Fourier components of the system’s transition matrix elements,

11 $$\langle {u}_{f}(t)|\gamma x|{u}_{i}(t)\rangle =\sum _{\ell }\,{{\rm{e}}}^{{\rm{i}}\ell \omega t}\,{V}_{fi}^{(\ell )},$$

and the numbers $N(\tilde{\omega })$ specify the thermal occupation of the phonon modes,

12 $$N(\tilde{\omega })=\{\begin{array}{ll}\frac{1}{\exp (\beta \hslash \tilde{\omega })-1} & ;\,\tilde{\omega } > 0\\ N(\,-\,\tilde{\omega })+1 & ;\,\tilde{\omega } < 0\end{array}$$

with *β* = 1/(*k*_B_*T*_bath_) encoding the inverse of the bath temperature *T*_bath_, invoking the Boltzmann constant *k*_B_. Observe that negative transition frequencies (10) correspond to processes during which the system looses energy to the bath so that a bath phonon is created, explaining the “+1” in the second case (12) which will turn out to be crucial. The third input required for evaluating the partial rates (9) is the spectral density $J(\tilde{\omega })$ of the oscillator bath; observe that the transition frequencies (10) enter into this density with their absolute value only.

Knowing the total rates (8), the quasistationary distribution {*p*_*n*_}_*n*_ _= __0,1,2,…_ of the Floquet-state occupation probabilities characterizing the steady state is obtained as solution to the master equation^39^

13 $$0=\sum _{m}\,({\Gamma }_{nm}{p}_{m}-{\Gamma }_{mn}{p}_{n}).$$

For the system (1) with system-bath coupling (7) the matrix Γ becomes tridiagonal, connecting neighboring Floquet states *m* = *n* ± 1 only. Moreover, for each such transition the ratio *r* of the “upward” rate Γ_*n*+1,*n*_ to the matching “downward” rate Γ_*n*,*n*+1_, namely,

14 $$\frac{{\Gamma }_{n+1,n}}{{\Gamma }_{n,n+1}}=r$$

becomes independent of the oscillator quantum number *n*. Therefore, the Floquet-state occupation probabilities for the quasistationary state are given by the geometric distribution

15 $${p}_{n}=(1-r)\,{r}^{n},$$

provided *r* < 1. If *r* > 1 the system does not reach a steady state, but keeps on climbing the oscillator ladder, its “upward” transitions being favored over the “downward” ones. Since the system’s quasienergies (6)are equidistant, one may introduce a quasitemperature *τ* by regarding *r* as a Boltzmann factor,

16 $$r=\exp (-\frac{\hslash \nu }{{k}_{{\rm{B}}}\tau }).$$

Positive quasitemperatures then characterize a steady state with *r* < 1; the smaller *r*, the lower *τ*. In contrast, negative *τ* signal quasithermal instability. Needless to say, we are considering a nonequilibrium system which does not possess a temperature in the sense of equilibrium thermodynamics; the quasitemperature simply serves as a convenient parameter for comparing the driving-engineered quasistationary state to the state that would be adopted in thermal equilibrium.

Now the evaluation of the general expression (9) leads to the explicit result^30^

17 $$r=\frac{{\sum }_{\ell }\,{|{v}^{(\ell )}|}^{2}\,N(\,+\,\nu \,+\ell \omega )\,J(|\nu +\ell \omega |)}{{\sum }_{\ell }\,{|{v}^{(\ell )}|}^{2}\,N(\,-\,\nu \,-\ell \omega )\,J(|\nu +\ell \omega |)},$$

where ${v}^{(\ell )}$ denote the Fourier coefficients of the periodic parts of the classical Floquet solutions (5),

18 $$v(t)=\sum _{\ell }\,{{\rm{e}}}^{{\rm{i}}\ell \omega t}\,{v}^{(\ell )}.$$

This representation (17) contains the heart of the Floquet-state cooling mechanism. Let us assume that the spectral density $J(\tilde{\omega })$ is particularly large at an upward transition with *positive* frequency $\tilde{\omega }=\nu +{\ell }_{1}\omega$ accompanied by a reasonably large squared Fourier coefficient ${|{v}^{({\ell }_{1})}|}^{2}$, but relatively small at all others, so that all contributions to the ratio (17) with $\ell \ne {\ell }_{1}$ may be neglected. In this case one has approximately

19 $$r\approx \frac{N(\nu +{\ell }_{1}\omega )}{N(\nu +{\ell }_{1}\omega )+1}=\exp [\,-\,\beta \hslash (\nu +{\ell }_{1}\omega )] < 1$$

by virtue of Eq. (12). The larger ${\ell }_{1}$ can be made, that is, the more sizeable Fourier coefficients are available, the smaller *r* can be reached. It needs to be stressed that both the Fourier coefficient labeled ${\ell }_{1}$ and the density of states drop out here. These quantities set the scale of the corresponding partial rate (9) and, hence, determine the time required for relaxing to the quasithermal steady state; if the Fourier coefficient picked out by the density of states should be small, this relaxation time may be quite long. Quite intriguingly, the geometric Floquet-state distribution implied by this approximate identity (19) looks as if the *driven nonequilibrium* system were mapped to an *undriven equilibrium* system characterized by a Boltzmann distribution with the actual temperature of the ambient bath, but with a “renormalized” effective level spacing $\hslash (\nu +{\ell }_{1}\omega )$ selected by the density of states, although this density itself does not figure in the end. It will be interesting to explore whether this particular feature exhibited by the present model is capable of generalization.

Here we stick to the idea of characterizing the quasistationary steady state of the driven system in terms of the quasitemperature introduced through Eq. (16). Evidently, the approximation (19)allows one to cover practically the entire interval 0 < *r* < 1, implying that the range of quasitemperatures accessible to the system is 0 < *τ*/*T*_bath_ < ∞. Thus, the quasitemperature may be quite different from the bath temperature *T*_bath_; in particular, the driven system can effectively be much colder than its environment.

## Results

In order to substantiate this key issue we now specify a Gaussian spectral density

20 $$J(\tilde{\omega })={J}_{0}\exp (-\frac{{(\tilde{\omega }-{\tilde{\omega }}_{0})}^{2}}{{(\Delta \tilde{\omega })}^{2}})$$

centered around a frequency ${\tilde{\omega }}_{0}$ with width $\Delta \tilde{\omega }$. The parameters entering into the Mathieu spring function (2) are chosen as ${\Omega }_{0}/\omega =\sqrt{2}$ and *Ω*_1_/*ω* = 1.0, giving the characteristic exponent *ν*/*ω* ≈ 1.387, only slightly down-shifted against the unperturbed oscillator frequency by the ac Stark effect^30^. Finally, the bath temperature is adjusted to $\hslash$*ω*/(*k*_B_*T*_bath_*) *= *β*$\hslash$*ω* = 0.1.

**Figure 1:**
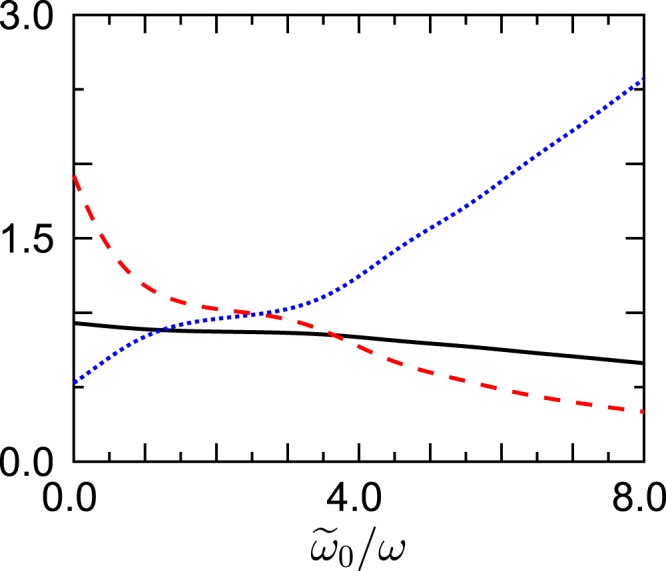
Ratio *r* (full line; black), scaled quasitemperature *τ*/*T*_bath_ (dashed line; red), and scaled occupation probability *p*_0_/*P*_0_ (dotted line; blue) of the Floquet state *n* = 0 vs. the center ${\tilde{\omega }}_{0}/\omega$ of a Gaussian spectral density (20) with large width $\Delta \tilde{\omega }/\omega =1.0$. The system parameters are ${\Omega }_{0}/\omega =\sqrt{2}$ and Ω_1_/*ω* = 1.0; the bath temperature has been set to *β*$\hslash$*ω* = 0.1.

In Fig. 1 we display the ratio *r*, the scaled quasitemperature *τ*/*T*_bath_, and the scaled population *p*_0_/*P*_0_ of the Floquet state *n* = 0, where *P*_0_ = 1 − exp(−*β*$\hslash$Ω_0_) is the thermal occupation of the oscillator ground state without periodic driving, attained when Ω_1_/*ω* = 0. Here we have chosen the width $\Delta \tilde{\omega }/\omega =1.0$ of the spectral density (20); data are plotted vs. its center ${\tilde{\omega }}_{0}/\omega$. We observe a steady decrease of *r* with increasing center frequency ${\tilde{\omega }}_{0}/\omega$, accompanied by the corresponding decrease of the quasitemperature, and a fairly significant increase of the population of the Floquet state *n* = 0, such that the latter exceeds the thermal equilibrium value by a factor of about 2.5 for ${\tilde{\omega }}_{0}/\omega \approx 8$. This finding already provides an encouraging verification of the mechanism underlying the approximation (19): Here the density (20) successively favors Floquet transition frequencies with ${\ell }_{1}=-\,1,0,1,2,\ldots \,$, but the Gaussian is still so wide that these transitions are not individually resolved.

**Figure 2:**
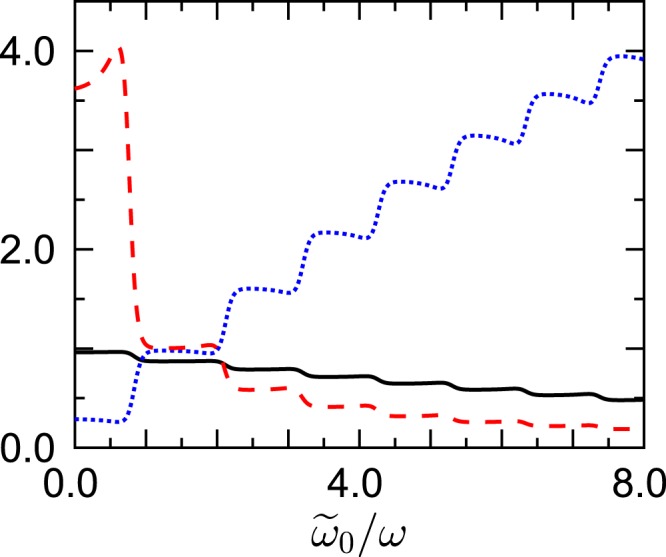
As Fig. 1, but for reduced width $\Delta \tilde{\omega }/\omega =0.316$. The sequence of plateaus is well captured by Eq. (19) with ${\ell }_{1}=-\,1,0,1,2,\ldots$.

This changes when the density width is reduced to $\Delta \tilde{\omega }/\omega =0.316$, while all other parameters are left unchanged, as shown in Fig. 2: Here the scaled *n* = 0-population features a series of well-developed plateaus with increasing center frequency which are explained with remarkable accuracy by Eq. (19), recalling *ν*/*ω* ≈ 1.387 and *β*$\hslash$*ω* = 0.1.

**Figure 3:**
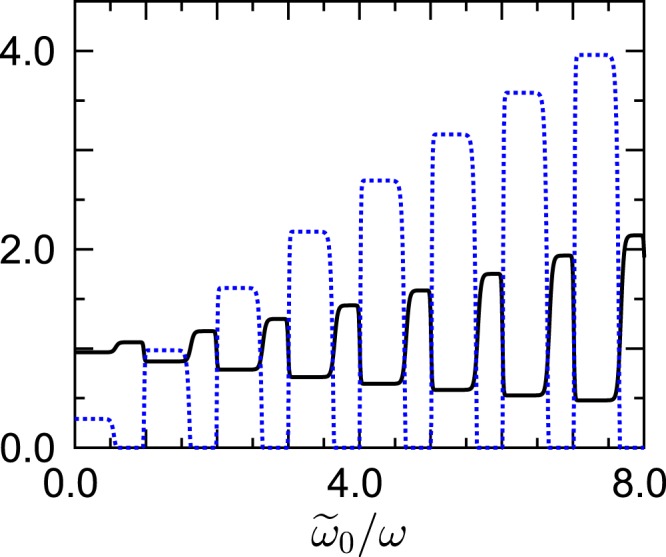
Ratio *r* (full line; black) and scaled occupation probability *p*_0_/*P*_0_ (dotted line; blue) of the Floquet state *n* = 0. All parameters are identical to those employed in Figs. 1 and 2, except for the density width which is reduced further to $\Delta \tilde{\omega }/\omega =0.1$ here. Plateau values of *p*_0_/*P*_0_ with *r* < 1 are governed by Eq. (19) with ${\ell }_{1}=-\,1,0,\ldots ,6$; intervals of quasithermal instability with *r* > 1 by Eq. (21) with ${\ell }_{2}=-\,2,-\,3,\ldots ,-\,9$. Observe that *p*_0_/*P*_0_ ≈ 4 for $7\lesssim {\tilde{\omega }}_{0}/\omega \lesssim 7.5$, corresponding to *τ*/*T*_bath_ ≈ 0.19.

A noteworthy phenomenon occurs when the density peak is made still sharper: Fig. 3 depicts both *r* and *p*_0_/*P*_0_ for $\Delta \tilde{\omega }/\omega =0.1$. Now one observes *two* plateau sequences, intervals of successively lower *r* and, hence, lower quasitemperatures allowing for higher *n* = 0-population alternating with intervals of successively higher *r*, indicating successively stronger quasithermal instability. For explaining this numerical finding one has to go back to the exact Eq. (17), and to appreciate the fact that only the absolute value of the Floquet transition frequencies enters into the density $J(|\tilde{\omega }|)$. Thus, when a peaked density such as described by Eq. (20) is appreciably large at a positive frequency $\nu +{\ell }_{1}\omega$, it may also be large at a *negative* frequency $\nu +{\ell }_{2}\omega$, then leading to

21 $$r\approx \frac{N(\,-\,\nu -{\ell }_{2}\omega )+1}{N(\,-\,\nu -{\ell }_{2}\omega )}=\exp [\,-\,\beta \hslash (\nu +{\ell }_{2}\omega )] > 1.$$

This is the key to understanding Fig. 3: The sequence of stable plateaus with *r* < 1 again is explained by Eq. (19) with ${\ell }_{1}=-\,1,0,\ldots 6$ to an accuracy on the sub-percent level, while the zones of quasithermal instability are governed by this Eq. (21) with ${\ell }_{2}=-\,2,-\,3,\ldots ,-\,9$. In principle, both kinds of processes have been competing already in the situation considered in Fig. 2, but there the “bad” processes have been overshadowed because of their smaller Fourier weights. It is only the narrow peak width employed in Fig. 3 which allows one to disentangle the “good” transitions from the “bad” ones. It deserves to be pointed out that one reaches a scaled *n* = 0-population *p*_0_/*P*_0_ ≈ 4 for $7\lesssim {\tilde{\omega }}_{0}/\omega \lesssim 7.5$, as corresponding to the scaled quasitemperature *τ*/*T*_bath_ ≈ 0.19. Thus, our present proof-of-principle study vindicates that Floquet-state cooling can be fairly effective. Among others, our results imply that an ideal Bose gas, stored in a parametrically driven oscillator trap, can condense into a macroscopically occupied single-particle Floquet state even if the ambient temperature is higher than the usual critical temperature. We also point out that that this novel type of “cooling by driving” is thermodynamically consistent: The non-equilibrium steady state is characterized by an energy flow which is always directed from the driven system into the bath, even if the quasitemperature of the former is lower than the actual temperature of the latter^30^.

## Discussion

The integrable model of the parametrically driven oscillator (1) features an unusually simple quasienergy spectrum (6) in its stability regime. Combined with a system-bath coupling of the natural form (7) it gives rise to a merely tridiagonal Floquet transition matrix (8) and therefore leads to the expression (17) which allows one to discuss the effect of the environment on the quasistationary state in an exceptionally transparent manner. Yet, the very essentials of the Floquet-state cooling mechanism — a rich Fourier spectrum of the Floquet transition matrix elements (11), the components of which may be addressed individually with suitable densities of states — will be present also in realistic, non-integrable periodically driven systems with dense pure point quasienergy spectra, even if their quasistationary states can no longer be characterized in terms of a quasitemperature. Moreover, for such chaotic systems it may no longer be feasible to assign meaningful quantum numbers to the Floquet states which depend on the parameters in a continuous manner, because their quasienergies exhibit a dense net of avoided crossings^10^; in particular, it may no longer be feasible to identify a “Floquet ground state” by continuity. Nonetheless, it will still be possible to select some Floquet state of interest, and to guide population into that state by means of suitable bath densities of states. Therefore, the mechanism that has been exemplified with the help of the model (1) is not restricted to that model, but fairly general. For these reasons we expect that Floquet-state cooling may find practical applications, *e.g*., with periodically driven solid-state systems interacting with a phonon bath predominantly at certain well-defined frequencies. One may also envision deliberate quasithermal engineering, amounting to the design of either favorable system environments, or of particular driving forms in order to enrich the Floquet system’s Fourier content.

The theoretical framework employed here, based on the golden rule-type rates (9), is equivalent to the standard Born-Markov approximation^38^, but a parametrically driven harmonic oscillator coupled to *N* bath oscillators constitutes an integrable system for any *N*^40^. Thus, it will be worthwhile to explore whether and how the findings reported in the present matter-of-principle study are recovered in the proper limit *N* → ∞ without invoking any approximation at all.

It is known that in many situations a periodic drive can be switched off adiabatically, such that the occupation probabilities of the Floquet states remain almost constant, even if the switch-off takes place within only a few driving cycles^41,42^. On the other hand, the time scales required for relaxing to the quasistationary distribution of Floquet-state occupation probabilities depend on the coupling to the bath which is weak by assumption. Therefore, under appropriate conditions it should be feasible to switch off the drive in an effectively adiabatic manner on times scales significantly shorter than the relaxation times after the quasistationary state has been reached, so that its Floquet-state occupation probabilities determine the occupation probabilities of the system’s proper energy eigenstates. This sketch might yield a blueprint for an actual cooling mechanism, providing higher-than-thermal ground-state populations.

## Data Availability

No datasets other than those plotted in Figs. 1–3 were generated or analysed during the current study.
