# Publisher Correction: Floquet-state cooling

**DOI:** 10.1038/s41598-020-60117-z

**Published:** 2020-02-19

**Authors:** Onno R. Diermann, Martin Holthaus

**Affiliations:** Institut für Physik, Carl von Ossietzky Universität, D-26111 Oldenburg, Germany

Correction to: *Scientific Reports* 10.1038/s41598-019-53877-w, published online 26 November 2019

This Article contains errors in the Reference section:

“12. Iadecola, T., Neupert, T. & Chamon, C. Classification of the Floquet statistical distribution for time-periodic open systems. Phys. Rev. B 91, 144301 (2015).”

should read:

“12. Liu, D.E. Classification of the Floquet statistical distribution for time-periodic open systems. Phys. Rev. B 91, 144301 (2015).”

“13. Th. Iadecola, T., Neupert & Chamon, C. Occupation of topological Floquet bands in open systems. Phys. Rev. B 91, 235133 (2015).”

should read:

“13. Iadecola, T., Neupert, T. & Chamon, C. Occupation of topological Floquet bands in open systems. Phys. Rev. B 91, 235133 (2015).”

“15. Vajna, S., Horovitz, B., Dora, B. & Zarand, G. Floquet theorem with open systems and its applications. Phys. Rev. A 93, 032121 (2016).”

should read:

“15. Dai, C.M., Shi, Z.C. & Yi, X.X. Floquet theorem with open systems and its applications. Phys. Rev. A 93, 032121 (2016).”

“16. Vajna, S., Horovitz, B., Doraand, B. & Zarand, G. Floquet topological phases coupled to environments and the induced photocurrent. Phys. Rev. B 94, 115145 (2016).”

should read:

“16. Vajna, S., Horovitz, B., Dora, B. & Zarand, G. Floquet topological phases coupled to environments and the induced photocurrent. Phys. Rev. B 94, 115145 (2016).”

